# A case of intussusception developed at the site of ileocolic anastomosis after laparoscopic right hemicolectomy

**DOI:** 10.1186/s12893-019-0539-z

**Published:** 2019-07-02

**Authors:** Tamuro Hayama, Yojiro Hashiguchi, Kohei Ohno, Yuka Okada, Kentaro Nemoto, Takahiro Yagi, Mitsuo Tsukamoto, Yoshihisa Fukushima, Tsuyoshi Ozawa, Ryu Shimada, Koichi Okamoto, Takeshi Tsuchiya, Keijiro Nozawa, Keiji Matsuda

**Affiliations:** 1 Department of Surgery, Teikyo University School of Medicine, 2-11-1 Kaga, Itabashi-ku, Tokyo, 183-8605 Japan; 2 Department of Surgery, National Defense Medical College, Tokorozawa, Japan

**Keywords:** Intussusception, Functional end-to-end anastomosis, Laparoscopic surgery

## Abstract

**Background:**

Intussusception is a relatively common condition seen in children. In comparison, adult intussusception is rare and usually occurs as a complication in patients with organic diseases. It is responsible for 1% of all bowel obstructions, in most of intussusceptions a malignant tumor is involved. Herein, we present an extremely unusual case of intussusception that occurred as a complication at the site of a functional end-to-end anastomosis.

**Case presentation:**

A 57-year-old female patient was diagnosed with tumors in the ascending and descending colon and was referred to our department. Laparoscopic hemicolectomy and laparoscopic descending colectomy were performed. The mechanical intestinal obstruction occurred on the 9th day postoperatively, and computed tomography scan revealed intussusception at the site of the ileocolic anastomosis. Endoscopic reduction was attempted, but the procedure was challenging. Surgery was then performed and revealed that the site of ileocolic anastomosis firmly adhered to the side wall and right retroperitoneum. However, the intestine in the oral side of the anastomosis was not fixed. Examination of the anastomotic site revealed that the ileum had passed through the anastomosis and prolapsed into the transverse colon. The ileocolic anastomosis was resected. End-to-end anastomosis was performed, and surgery was then completed. No neoplastic lesions were observed in the resected tissue of the lead point of intussusception. The postoperative clinical course was favorable, and the patient was discharged on the 11th day after the second round of surgery.

**Conclusions:**

There are no reports the anastomosis is involved as part of the intussception, as observed in the present case. Intussusception should thus be considered as one of the causes of postoperative mechanical intestinal obstruction.

## Background

Intussusception in adults is rare, and usually occurs as a complication in patients with organic diseases. We report a case of intussusception that occurred as a complication at the site of a functional end-to-end anastomosis.

## Case presentation

A 57-year-old female patient presented with a chief complaint of abnormal findings upon medical examination. She had no significant lifestyle/family history or medical history. Regarding her history of present illness, the patient was referred to our department after undergoing a computed tomography (CT) scan in September 2017, which revealed a tumor in the descending colon.

Hematological analysis revealed the following results: White blood cell count, 7900/μl; hemoglobin level, 12.6 g/dL; platelet count, 28.4 × 10^4^/μl; carcinoembryonic antigen level, 3.3 ng/mL; and CA 19–9 level, 11.1 U/mL. The results were not significant.

Lower gastrointestinal endoscopy findings revealed a 25-mm type 0-Is lesion observed in the ascending colon and a 30-mm type Is lesion in the descending colon. In the abdominal contrast-enhanced CT findings, the tumors could not be located, and lymph node, lung, or liver metastases were not observed. Regarding histopathological findings, biopsy results revealed that the tumor in the ascending colon was a high-grade adenoma and the tumor in the descending colon was a moderately differentiated adenocarcinoma. Endoscopic tumor resection was not possible due to the difficulty in maneuvering the endoscope in the ascending and descending colon. Thus, surgery was considered.

**Fig. 1:**
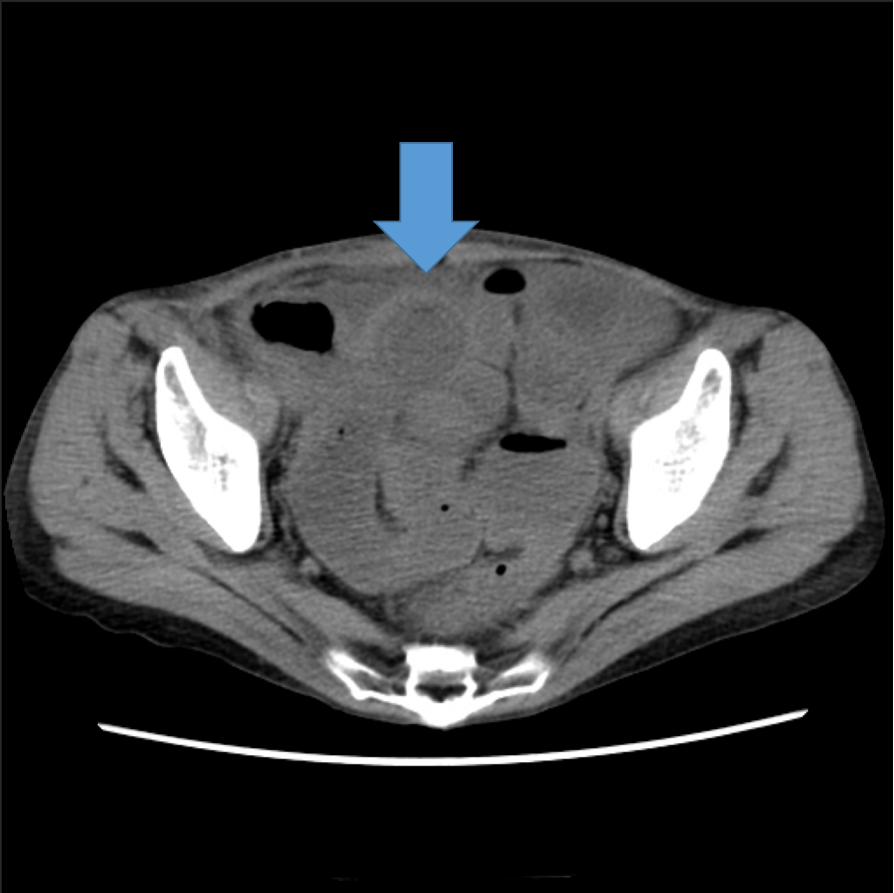
Abdominal computed tomography scan. Intestinal fluids are accumulated in the small intestine, and the target sign can be observed (arrow)

**Fig. 2:**
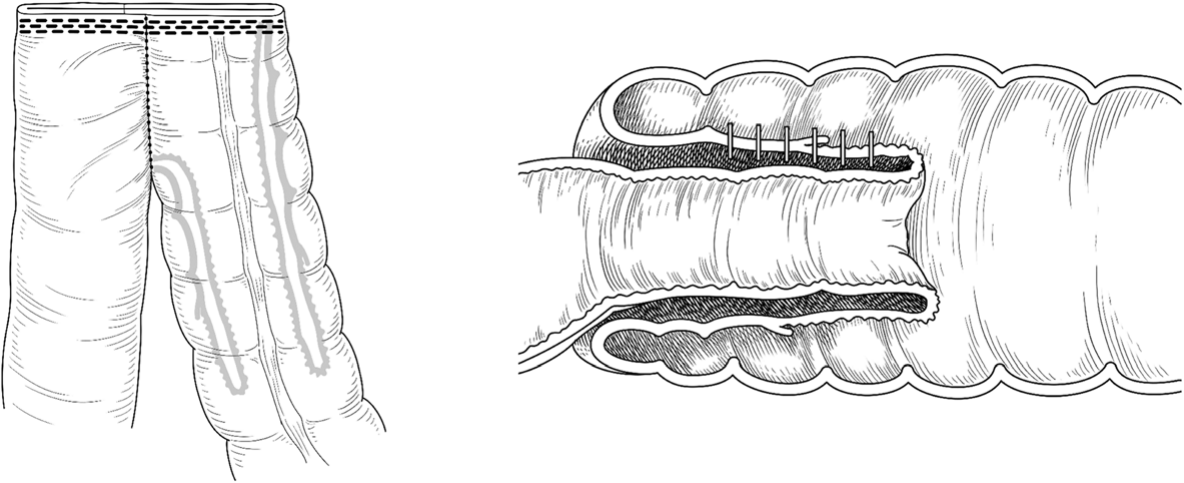
Diagram showing differences anastomosis intussusception

**Fig. 3:**
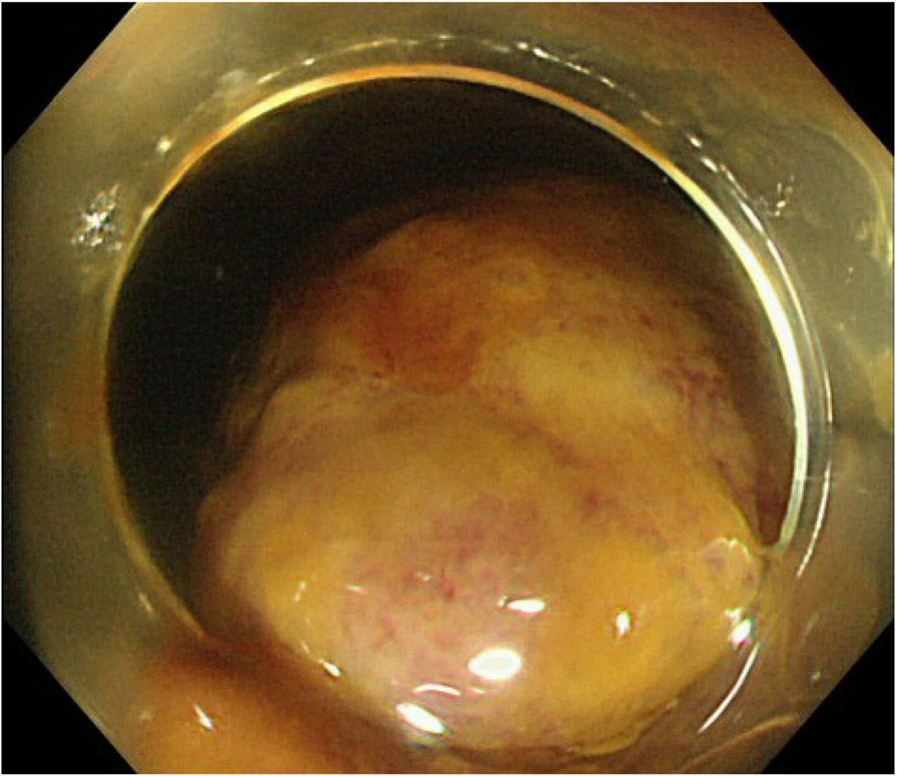
Endoscopic image. Intussusception was observed, and the mucous membrane of the ileum was reddened

In the first round of surgery, surgery was initiated with five ports. No ascites, peritoneal dissemination, or liver metastases were observed. The ascending colon polyp was adenoma, but there was a polyp near the ileocecal valve. Therefore, it was difficult to resect the ascending colon and we chosed the right hemicolectomy. The descending colon polyp was adenocarcinoma on biopsy. However, we diagnosed intramucosal cancer and performed descending colon resection. Laparoscopic resection of the descending colon and right hemicolectomy was performed according to standard procedures. There were two functional end-to-end anastomoses. Regarding the clinical course after the first round of surgery, mechanical intestinal obstruction occurred on the 9th day postoperatively, and CT scan showed that intussusception occurred from the functional end-to-end anastomosis (Figs. 1 and 2). An attempt at endoscopic reduction was unsuccessful (Fig. 3), and open surgery was considered on the 16th day after the first round of surgery.

**Fig. 4:**
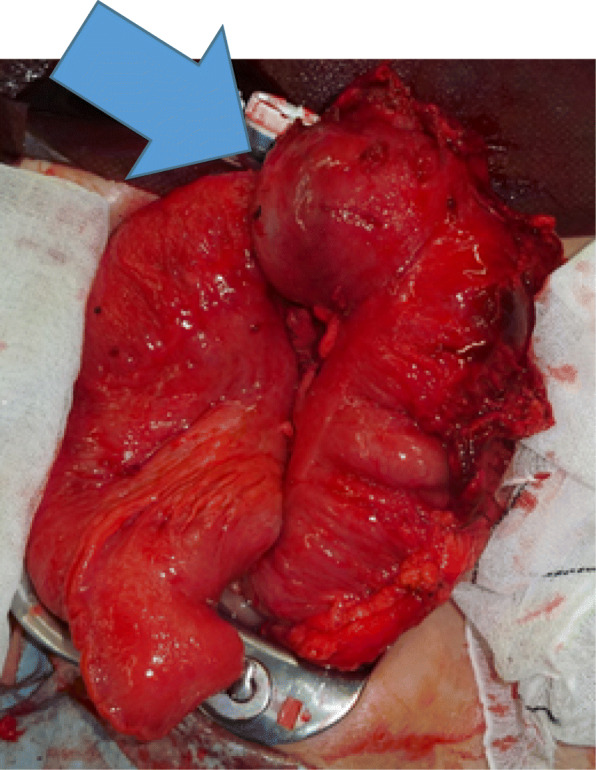
Intraoperative image. Anastomosis is prolapsed in the direction of the arrow (transverse colon)

**Fig. 5:**
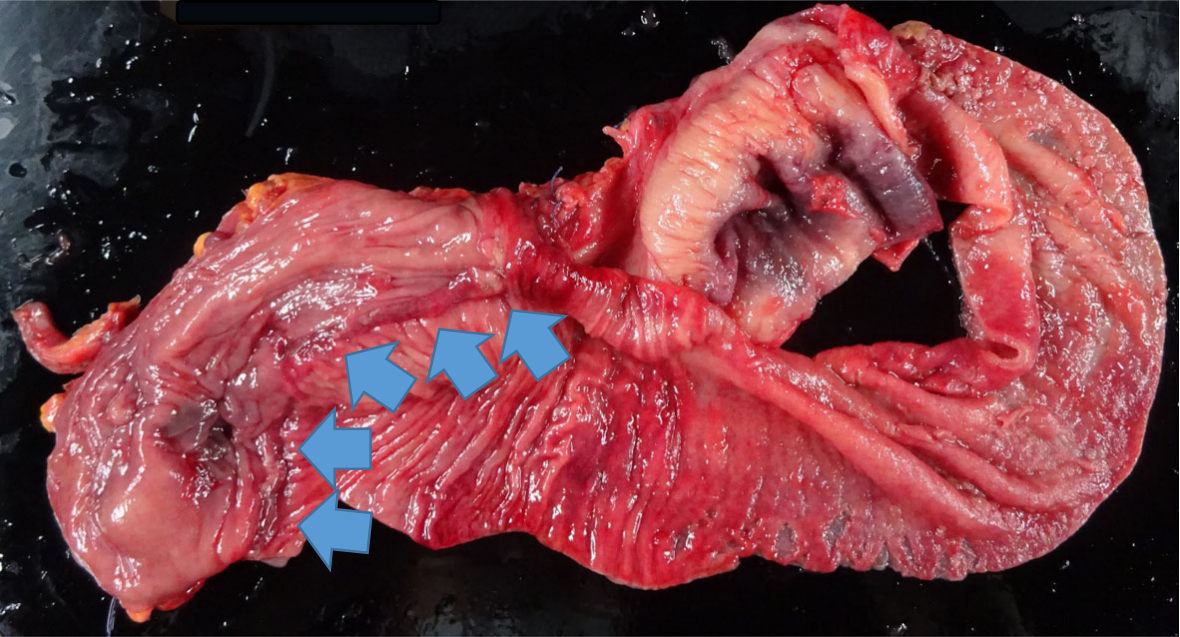
Photograph of the specimen. Anastomosis is indicated with the arrow. The large intestine is on the left side, and the ileum is on the right side

In the second round of surgery, when midline laparotomy was performed, the site of the ileocolic anastomosis was found to be firmly adhered to the side wall and right retroperitoneum. Because the staple used for anastomosis may have adhered to the peritoneum. The intestines in the proximal side of the anastomosis were not fixed. Examination of the anastomosis revealed that the ileum had passed through the anastomosis and entered the transverse colon (Fig. 4). Although manual reduction was attempted, manipulation was challenging. The ileocolic anastomosis was resected. End-to-end anastomosis was performed, and surgery was completed. Histopathological findings revealed no lesions in the resected intussusceptum (Fig. 5). Regarding the clinical course after the second round of surgery, the postoperative clinical course was favorable, and the patient was discharged on the 10th day after the second surgery.

## Discussion and conclusions

Intussusception in adults is rare [1]. Over 90% of adult cases are associated with a specific etiology; approximately 19–42% are caused by malignant tumors, and 22–41% are attributed to benign tumors [2]. Approximately 90% of intussusception cases in adults are associated with organic diseases, and only 1–4% occur at the anastomotic site [1, 3, 4]. The pathogenetical mechanisms of postoperative intussusception are not well-understood due to its rarity. However, several hypotheses including prolonged postoperative ileus, extensive non-gentle handling and drying of intestines at primary surgery, postoperative radiation or chemotherapy have been proposed. [5] Although intestinal or abdominal adhesion is assumed to cause postoperative intussusception, mild intraperitoneal adhesion was observed in the present case.

Intussusception caused by functional end-to-end anastomosis is extremely rare. We searched the PubMed database for articles published between 1970 and December 2017 using “intussusception,” “anastomosis,” and “right hemicolectomy” as keywords. However, we did not find any relevant articles. “Intussusception” and “functional end-to-end Anastomosis” did not hit any human case reports.

The chief complaints only few symptoms that characterize the condition are observed, and the clinical course often varies [6]. Diagnosis of postoperative intussusception is difficult that ileus is a far more common postoperative morbidity, presenting with symptoms similar to obstruction, including nausea, vomiting, abdominal distention, and obstipation. Further complicating the clinical picture in these patients is postoperative narcotics masking symptoms and reliable physical exams and the known negative effects of opiates on intestinal peristalsis. Postoperative ileus usually lasts on average 3 to 5 days, but can last much longer [7]. Therefore, it is challenging to establish a diagnosis based on only the clinical condition. However, there are characteristic findings that can be observed during diagnostic imaging. In abdominal ultrasonography, the donut sign, target sign, or multiple concentric ring sign, all of which are characteristic images, can be observed. In an abdominal CT scan, the multiple concentric ring sign, which comprises concentric circular repeats of high and low density layers, can be observed. These characteristic findings can easily facilitate the diagnosis via abdominal CT scan [8]. In the present case, the target sign was observed.

Intussusception is first treated with a barium enema or endoscopic reduction [9, 10]. In the present case, although endoscopic reduction was attempted, the procedure was challenging. In cases where endoscopic reduction is difficult, open surgery is then performed. When the intestine is mobile, as it is in cases with mobile cecum, surgical reduction procedures that do not involve intestinal resection but only reduction and fixation must be considered. Enterectomy should be performed if a tumor is suspected in the lead point of the intussusception.

Currently, idiopathic intussusception is believed to occur when a stimulus causes the circular muscle of the intestine to contract convulsively and enter the relaxed intestine that is contiguous to it in the anal side [11]. Elechi et al. have reported that a mobile cecum can be a causative factor of intussusception [12]. If hemicolectomy is performed, as in the present case, then the site of anastomosis becomes mobile. Excessive peristalsis correlates with the onset of intussusception. Contractions in the left side of the colon can be classified as “short duration” (propagating at 4–6 cm/min and lasting for 10 s) or “long duration” (propagating at 0.5–2.0 cm/min and lasting for approximately 1 min) and are stimulated by physical factors such as the gastrocolic reflex. [3] However, the contractions in the right side of the colon differ from those in the left side, and they have the characteristics of mass peristalsis or vertical contraction. Mass peristalsis in the right side of the colon propagates at 12–180 cm/min and lasts for 1 min. The contractions in the right side are approximately two to three times stronger than those on the left side. Moreover, mass peristalsis in the right side of the colon can occur without requiring physical stimulation [3]. In other words, the right side of the colon has stronger peristalsis even in a physiological environment. In addition, its segments are longer, and there are relatively more cases in which it is mobile. Thus, it is relatively easy for intussusception to occur in that site (in comparison to other sites in the digestive tract). In the present case, although it was very difficult to identify the cause, it may be possible that the ileum advanced to the anal side and occured with intussusception because the peristalsis of the ileum is stronger than the colon. Relationship was not clear that intussusception occur as a complication owing to the location of the anastomosis in the right side of the colon and its high mobility, in addition to the peristaltic contractions in the right side.

As a surgery to prevent recurrence, there are methods to prevent invasion of the ileum into the colon by fixing the intestine to the abdominal wall, and to suppress recurrence by promoting postoperative adhesion [13–15]. We did not do it in this case, but we need to consider it.

Intussusceptions in adults are often caused by tumors, and they are rarely caused by other factors. To the best of our knowledge, no studies have reported cases of anastomosis as a cause of intussusception, as reported in the present case. Our case indicated that intussusception should be considered as one of the causes of postoperative mechanical intestinal obstruction.

## Data Availability

All available data supporting our findings are listed in the tables which are shown in the manuscript. Thus, our conclusions can be ascertained from the published data.

## Abbreviation

CT: computed tomography
